# Dietary Polyphenols: Promising Adjuvants for Colorectal Cancer Therapies

**DOI:** 10.3390/cancers13184499

**Published:** 2021-09-07

**Authors:** Laura Bracci, Alessia Fabbri, Manuela Del Cornò, Lucia Conti

**Affiliations:** 1 Department of Oncology and Molecular Medicine, Istituto Superiore di Sanità, Viale Regina Elena 299, 00161 Rome, Italy; 2 Department of Cardiovascular, Endocrine-Metabolic Diseases and Aging, Istituto Superiore di Sanità, Viale Regina Elena 299, 00161 Rome, Italy; alessia.fabbri@iss.it; 3 Center for Gender-Specific Medicine, Istituto Superiore di Sanità, Viale Regina Elena 299, 00161 Rome, Italy; manuela.delcorno@iss.it

**Keywords:** polyphenols, colorectal cancer, anticancer drug, preclinical study, clinical study, combination therapy

## Abstract

**Simple Summary:**

Colorectal cancer is a leading cause of death worldwide. Despite the development of novel surgical and therapeutic strategies, 50% of patients relapse after treatment. Therapy failure, due to low efficacy, adverse effects and drug resistance, is thus a major concern. The idea of combining standard therapy with non-toxic bioactive natural compounds is a recent topic in cancer research and aims to increase the efficacy of current antitumor therapies while reducing drug toxicity and adverse effects. In recent years, several studies have explored the capacity of polyphenols, dietary bioactive compounds enriched in fruit and vegetables, to act as adjuvants to improve colorectal cancer therapy. In the present review, we discuss these studies, highlighting the mechanisms underlying the adjuvant effect, and bring out the potential of this novel therapeutic approach as well as the critical issues related to clinical application.

**Abstract:**

Colorectal cancer (CRC) is a major cancer type and a leading cause of death worldwide. Despite advances in therapeutic management, the current medical treatments are not sufficient to control metastatic disease. Treatment-related adverse effects and drug resistance strongly contribute to therapy failure and tumor recurrence. Combination therapy, involving cytotoxic treatments and non-toxic natural compounds, is arousing great interest as a promising more effective and safer alternative. Polyphenols, a heterogeneous group of bioactive dietary compounds mainly found in fruit and vegetables, have received great attention for their capacity to modulate various molecular pathways active in cancer cells and to affect host anticancer response. This review provides a summary of the most recent (i.e., since 2016) preclinical and clinical studies using polyphenols as adjuvants for CRC therapies. These studies highlight the beneficial effects of dietary polyphenols in combination with cytotoxic drugs or irradiation on both therapy outcome and drug resistance. Despite substantial preclinical evidence, data from a few pilot clinical trials are available to date with promising but still inconclusive results. Larger randomized controlled studies and polyphenol formulations with improved bioavailability are needed to translate the research progress into clinical applications and definitively prove the added value of these molecules in CRC management.

## 1. Introduction

Colorectal cancer (CRC) is the third most common and the second deadliest cancer in both males and females, with more than 900,000 deaths annually worldwide [1]. Developed countries are at the highest risk of CRC development, thus suggesting a causal relationship with lifestyle. Indeed, CRC is a multifactorial disease with genetic as well as environmental etiology. Infections, chronic inflammation, obesity and eating habits are among the main risk factors for CRC. Over the past two decades, the survival rates of patients diagnosed with early stage CRC have significantly improved. However, more than 50% of patients are diagnosed with metastatic disease, for whom the five-year survival rate is approximately 10% [2]. The treatment of CRC mostly relies on surgery, which is often accompanied by pre-operative (neo-adjuvant) or post-operative (adjuvant) chemotherapy and radiotherapy. Treatment-related, severe adverse effects and/or drug resistance strongly contribute to therapy failure and to tumor recurrence and progression. Thus, there is an urgent need to explore novel non-toxic compounds that can be used in combination with standard treatments with the aim of reducing side effects, preventing resistance and improving tumor response to the current therapeutic options. Accumulating evidence suggests that natural bioactive compounds, including dietary polyphenols, can exert chemopreventive as well as direct antitumor activities by regulating several molecular targets involved in cancer cell survival, proliferation and invasion, as well as in angiogenesis [3,4] and immunomodulation [5]. Interestingly, some natural compounds displayed additive or synergistic activities when combined with conventional cytotoxic therapies in CRC, thus opening the way to clinical translation [6].

In the present review, we provide a brief overview of the role of dietary polyphenols in CRC pathogenesis and treatment and summarize recent preclinical and clinical studies that have explored the capacity of these compounds to act as adjuvants to CRC therapies. Finally, we discuss the relevance of these studies in filling some gaps in CRC management and some bottlenecks that may hamper the clinical translation of results.

## 2. Pathogenesis and Clinical Management of Colorectal Cancer

A growing body of evidence indicates that CRC is a multi-factorial disease, with a variety of factors playing a role in its pathogenesis. Heritable factors account for approximately 35% of CRC risk [7]. Thus, more than 60% of CRC cases are estimated to be caused by modifiable risk factors, such as smoking, heavy alcohol consumption, obesity, unhealthy eating habits, physical inactivity, infections and chronic inflammation [8]. Gut microbiota alterations can also contribute to disease [9]. Chromosomal instability (CIN), microsatellite instability (MSI) and CpG island methylation are the three main pathways involved in colorectal carcinogenesis [10]. Although the underlying genetic alterations have been well characterized in CRC, the interplay between cancerous or even pre-cancerous cells and their microenvironment (i.e., immune cells, cancer-associated fibroblast, gut microbiota) greatly contributes to tumor development and progression [10].

At the time of diagnosis, approximately 80% of CRC cases are localized, whereas 20% have already metastasized into distant sites. Surgical resection remains the only curative option for colon and rectal cancers at all stages. Adjuvant chemotherapy, mainly FOLFOX (5-fluorouracil 5-FU, folinic acid and oxaliplatin OXA) or FOLFIRI (5-FU, folinic acid and irinotecan IRI), can help to eradicate the micro-metastases potentially occurring at the site of surgery, whereas for locally advanced tumors, neoadjuvant chemotherapy (mainly 5-FU and capecitabine) is indicated. Furthermore, chemoradiation is often required for locally advanced rectal cancer after surgical removal [11]. While chemotherapy is relatively effective in the early stages of the disease, the response rate in metastatic CRC (mCRC) is only 10–15% [12]. A combination of standard chemotherapy with more specific targeted therapies (aimed at blocking growth factor receptors or angiogenic factors) for molecularly defined mCRC has considerably improved survival but has also generated uncertainty about how to identify the optimal sequence and combination [13]. Recently, regorafenib (a multiple tyrosine-kinase inhibitor) has been approved for all patients with refractory mCRC not responding to previous treatments. Moreover, novel promising immunotherapeutic strategies have been authorized for patients bearing microsatellite unstable mCRC characterized by a high mutation burden, which, however, accounts for only a small proportion of patients [14]. In any case, about 50% of CRC patients will develop recurrent disease due to primary or acquired resistance [15]. The mechanisms that contribute to drug resistance include the mutations of drug targets, the inactivation of apoptotic pathways, enhanced DNA damage repair and alterations in drug metabolism [16]. The major contributors to drug resistance in CRC are cancer stem cells (CSC), a rare subpopulation of cancer cells endowed with self-renewal properties, unlimited cell division capability and differentiation potential [17]. The combined administration of agents with non-overlapping mechanisms of action and/or different cell targets and/or synergistic effects may be an encouraging strategy to increase treatment efficacy and to reduce the side effects associated with conventional treatments (i.e., neutropenia, diarrhea and gastro-, neuro- and nephro-toxicity, among others). In this respect, natural compounds endowed with antitumor effects and low toxicity could represent promising candidates for a combination to be further investigated.

## 3. Dietary Polyphenols

### 3.1. Classification of Polyphenols and Dietary Sources

Polyphenols comprise a large and heterogeneous group of phytochemicals containing one or more phenol rings [18]. Depending on the number of phenol rings that they contain and the structural elements that bind these rings to each other, polyphenols are categorized into several classes and subclasses. The main groups are flavonoids, phenolic acids, phenolic alcohols, stilbenes and lignans (see http://phenol-explorer.eu/compounds/classification for an update classification (accessed on 5 June 2021)).

Polyphenols come mainly from fruits, vegetables, whole grains and beverages (fruit juice, red wine, tea and coffee) [19]. Some of them are specifically present in particular food (flavanols in chocolate, flavanones in citrus fruit, isoflavones in soya and phlorizin in apples), whereas others, such as quercetin, are ubiquitously found in all plant products [19]. Generally, food contains complex mixtures of polyphenols, although most of them in the form of esters, glycosides, or polymers cannot be absorbed in the native form. Indeed, after ingestion, polyphenols generate several metabolites that reach cells and tissues and that are chemically, biologically and, in many instances, functionally different from the original dietary form, rendering the identification of active compounds extremely difficult [20]. These modifications also affect polyphenol bioavailability. The term ’bioavailability’ means the fraction of an ingested nutrient or compound that reaches the systemic circulation and the specific sites where it can exert its biological action. It is worth noting that bioavailability appears to differ greatly among various polyphenols. Consequently, it is more important to know how much of a polyphenol is bioavailable than how much of a nutrient is present in a specific food or dietary supplement. In this regard, the role of the gut microbiota in mediating polyphenol biotransformation and bioavailability is rapidly emerging [21], as well as the capacity of polyphenol-rich diets to enrich specific microbial species and increase microbial diversity [22]. In conclusion, the biological activity depends especially on the amount of polyphenols and their metabolites accumulated in target tissues, suggesting that the physiological in vivo context in which dietary polyphenols exert their influence is undoubtedly much more complex than that available from an in vitro system [23]. The same compound might show strong activity in vitro but could have little biological activity in vivo if little or none of the compound reaches the target tissues [24].

### 3.2. Biological Properties of Dietary Polyphenols

Accumulating evidence from epidemiological and observational studies has suggested possible associations between polyphenol intake and the risk of certain cancer incidence and mortality [25,26,27], while laboratory studies have supported the anti-inflammatory [28], antioxidant [29,30] and immunomodulatory activities [5,28] of natural polyphenols. In addition, evidence is emerging on the activity of dietary polyphenols as modulators of the colonic microbial population composition and activity [31]. Indeed, polyphenols and their derivatives are now recognized as one of the main families of natural compounds expected to be used as dietary factors to prevent chronic diseases [32].

Specifically, polyphenols inactivate NF-κB and modulate crucial cell signaling pathways involved in inflammation and cancer, such as MAPK and PI3K/Akt pathways [28]. They can suppress toll-like receptors (TLRs) and pro-inflammatory gene expression [33]. Their antioxidant activity and ability to inhibit enzymes involved in the production of eicosanoids also contribute to their anti-inflammation properties [28]. They inhibit certain enzymes involved in reactive oxygen species (ROS) production, such as xanthine oxidase [34,35,36] and NADPH oxidase (NOX) [37,38], while they upregulate other endogenous antioxidant enzymes, such as superoxide dismutase (SOD), catalase and glutathione peroxidase. Furthermore, the ability of polyphenols to reduce the release of arachidonic acid, prostaglandins and leukotrienes is considered one of their most important anti-inflammatory mechanisms [28]. In addition, the modulation of cytokines and chemokines is one of many common mechanisms by which polyphenols exert their immunomodulatory effects. Indeed, in vivo and in vitro studies demonstrate that polyphenols affect the biology of myeloid cells, macrophages and dendritic cells, as well as T cells and NK cells [33,39], by inhibiting multiple key regulators of the inflammatory response, such as TNF-α, IL-1β and IL-6 [40,41].

The effects of these biologically active compounds on the immune system are associated with extended health benefits for different chronic inflammatory diseases [42,43], including cancer [19,25], diabetes [44], obesity and inflammation-related diseases [45,46], neurodegenerative disorders and cardiovascular diseases [47].

### 3.3. Role of Polyphenols in Colorectal Cancer Prevention and Treatment

There is convincing evidence that Western dietary patterns, characterized by a high intake of red and processed meat and a low intake of fruit and vegetables, increase CRC risk (https://www.wcrf.org/wp-content/uploads/2021/02/Colorectal-cancer-report.pdf (accessed on 15 June 2021)) [48]. Conversely, adherence to the Mediterranean diet (MD), enriched in fruit, vegetables and fiber-containing foods, has been inversely correlated with both the risk of developing CRC and CRC-related mortality [49,50]. Accumulating epidemiological evidence indicates that MD can also improve prognosis and the quality of life of subjects already diagnosed with CRC, thus arguing for a role of diet and its components in both primary and tertiary CRC prevention [51].

Polyphenols are widely represented in the MD, and a higher adherence to the MD was shown to result in a higher polyphenol intake and an increased colonic expression of polyphenol-derived metabolites [52]. These dietary components have received great attention in CRC research for their chemopreventive and direct anticancer activities [31]. The antitumor effects of several compounds, belonging to the main groups of polyphenols, have been investigated in in vitro studies with cancer cells and in preclinical animal models. Furthermore, some data have also been produced regarding their application as anticancer compounds in clinical trials, although the vast majority of these studies are still in progress [53]. Specifically, curcumin, either as a free compound or in the form of nanoformulations, has been shown to display chemopreventive and anticancer activities against CRC by modulating multiple signaling pathways leading to the induction of cell cycle arrest and apoptosis and the downregulation of epidermal growth factor receptor expression [54]. Many studies have also proved the chemopreventive role of resveratrol in CRC [55]. This compound was reported to trigger apoptosis in tumor cells and to suppress colorectal tumorigenesis and inflammation in murine models [55].

Among flavonoids, quercetin and genistein were shown to affect a number of molecular pathways involved in colorectal tumorigenesis and tumor progression, including cell cycle, proliferation, apoptosis and oncogene expression, and to have a therapeutic effect in CRC preclinical models [56]. Conversely, the anthocyanin-mediated inhibition of colon cancer formation and growth in mouse models was reported to occur mainly via the suppression of inflammation and the regulation of angiogenesis [57].

The multifactorial nature of CRC highlights the need for multi-targeted approaches offering a more effective alternative to the current therapeutic options. Evidence is emerging that conventional chemotherapy in CRC significantly benefits from combined treatment with natural products such as polyphenols [58]. The promising role of polyphenols as sensitizers of standard chemo/radiotherapies and targeted therapies, which is discussed in detail in the following sections, paves the way for new combined strategies able to minimize the toxicity and side effects of conventional treatments and to reduce the risk of tumor recurrence.

## 4. Dietary Polyphenols as Adjuvants in Colorectal Cancer Therapy

### 4.1. Beneficial Effects of Polyphenols on Colorectal Cancer Treatment: Preclinical Evidence

The idea to use polyphenols in combination therapy has received great attention in recent decades, and a wealth of preclinical studies have been performed in order to analyze the effectiveness of combination and the mechanisms at the basis of their beneficial activity [58]. Here, we provide an update of the most recent literature on the effects of the most common dietary polyphenols on standard and experimental CRC treatments (summarized in Table 1).

#### 4.1.1. Curcumin

Among dietary polyphenols, curcumin is certainly the most studied for combined therapy against CRC. Curcumin’s anticancer activity has been analyzed in association with different chemotherapeutic drugs currently used for CRC management both in vitro and in vivo. Whatever the cell line used, curcumin showed a synergistic effect with the drug in terms of cell viability. In particular, a considerable reduction in the proliferative and migratory capabilities and in the apoptotic rates of the metastatic cell line SW620 was observed when 5-FU was combined with curcumin [59]. In this context, curcumin significantly inhibited phosphoERK (pERK) signaling and downregulated L1 expression, a cell adhesion molecule correlated with poor prognosis and metastasis. In addition, curcumin pre-treatment could significantly augment the cytotoxicity of 5-FU on HCT116 and HT29 cell lines and in HCT116 xenografts through the inhibition of autophagy [60], a cellular adaptation mechanism issued by tumor cells to counteract the cellular stress induced by chemotherapy [91]. In combination with IRI, curcumin induced cell cycle arrest and apoptosis in part mediated by ROS generation and the activation of endoplasmic reticulum (ER) stress in LoVo and HT29 cell lines [61]. Interestingly, on IRI-resistant LoVo cells, curcumin reduced the expression levels of CSC identification markers and significantly attenuated chemoresistance through the induction of apoptosis of CSC among colon cancer cells [62].

The ability of curcumin to reverse chemoresistance has also been described toward OXA [16]. Long-term application of the drug leads to toxic side effects and resistance, the major cause of therapy failure. In this context, curcumin increased the apoptotic rate of chemoresistant CRC and significantly inhibited its migration and invasion potential. In addition, Yin and coworkers [63] described an increase in caspase 3 activity and a decrease in the migratory ability of chemoresistant CRC cells through dampening the TGFβ/Smad2/3 signaling pathway both in vitro and in vivo. Han and coworkers [64], on the other hand, reported that curcumin can reverse drug resistance in the HCT116 cell line through its effects on the miRNA-mediated regulation of ERCC1, a key protein of the nucleotide excision repair (NER) system [92], with alterations in DNA repair ability being one of the main mechanisms of drug resistance. In another study, human CRC cell lines containing either mutated or wild-type KRAS were treated with regorafenib, a multiple kinase inhibitor, in combination with curcumin [65]. The addition of curcumin to regorafenib augmented apoptosis and autophagy rates in HCT116 (KRAS mutant) but not in HT-29 cells (KRAS wild-type), thus suggesting that curcumin functions as a MEK inhibitor to induce a synthetic lethal effect on KRAS-mutant CRC cells receiving the targeted drug regorafenib. In another study, the exposure of CT26-implanted mice to curcumin (50 mg/kg for 5 days, oral) and to the PDE5 inhibitor sildenafil (Viagra^®^) significantly impaired tumor growth and decreased the expression of PD-L1, PD-L2 [66]. The therapeutic effect was further enhanced by the administration of the anti-PD-1 antibody. In the same study, curcumin + sildenafil proved effective in combination with a clinically relevant concentration of 5-FU and caused further activation of DNA damage signaling, metabolic stress signaling and ER stress signaling [66]. Knocking down ataxia telangiectasia mutated (ATM), AMP-dependent protein kinase-α (AMPKα), eukaryotic initiation factor 2-α (eIF2α) or LC3-associated phagocytosis (LAP) significantly reduced the lethality of the combined treatment (curcumin + sildenafil + 5-FU) [66], confirming the involvement of the above-mentioned pathways. In another study, the capability of curcumin to sensitize CRC cell lines to gemcitabine (GEM) was investigated in patient-derived cell lines with different molecular characteristics (CpG island methylator phenotype, CIN and MSI). Treatment with curcumin plus GEM induced up to 70% biomass reduction in MSI + cell lines and a fivefold induction of ATM and cyclin-dependent kinase inhibitor 2 (CDKN2) [67]. A coculture of tumor and immune cells revealed the stimulation of immune-based cytotoxicity by curcumin in combination with either GEM or the IDO inhibitor indoximod [67].

Although curcumin is one of the most effective phytochemicals, its water solubility, metabolic instability and poor bioavailability limit its clinical application [93]. Thus, curcumin analogs have been synthesized, and some of them have been tested in vitro. Zhao and coworkers [69] analyzed the combined effects of dimethoxycurcumin (DMC), a lipophilic analog of curcumin, with 5-FU in SW480 and SW620 cell lines, describing an additive antitumor effect in both cell lines. This effect was closely related to cell cycle arrest and apoptosis induction as well as to an increase in ROS production, ER expansion and a decrease in mitochondrial membrane potential. These results are particularly important since DMC has an increased potential to induce colon cancer cell apoptosis, is less toxic to normal cells and possesses a higher bioactivity compared to curcumin [94]. In the same vein, prenylated curcumins that are semisynthetic curcumin derivatives have shown promising results in in vitro studies of combination therapy. In particular, gercumin exhibited a synergistic effect when tested in combination with FOLFOX, suppressing the growth of cancer cells with a potency similar to that of curcumin. Moreover, one of the combinations tested was also effective at suppressing colonosphere formation [70]. In mice implanted with CT26 tumor cells, oral administration of a nano-formulation of curcumin and resveratrol (300 mg/kg every 2 days for 2 weeks) in combination with modulated electro-hyperthermia (mEHT) treatment significantly suppressed tumor growth and triggered host immunity by recruiting T cells and F4/80+ macrophages into the tumor mass [71].

Radiotherapy is one of the treatments for CRC. It is important to underline that polyphenol effects have also been tested in combination with ionizing irradiation (IR). A combined treatment of curcumin (20 mg/kg, intra-peritoneal (i.p.)) and IR (10 Gy) resulted in significantly greater tumor growth inhibition and apoptosis compared to IR treatment alone [68]. Curcumin sensitized cancer cells to IR by altering the expression of DNA repair-related genes, including DNA ligase IV (LIG4), X-ray repair cross complementing 5 (XRCC5) and polynucleotide kinase/phosphatase (PNK).

#### 4.1.2. Resveratrol

One of the best known polyphenols is resveratrol, a naturally occurring plant antibiotic found in various plants, nuts and fruits and especially abundant in grapes and red wine [95]. Previous studies indicated that resveratrol potentiates the cytotoxic properties of doxorubicin (DOX), a widely used chemotherapy due to its efficacy against a wide range of cancers, via downregulation of the MDR1 gene and P-glycoprotein (P-gp) inhibition [96]. In CRC, resveratrol has been shown to suppress TNF-β-induced tumor metastasis and to chemosensitize CRC cells to 5-FU in 3D alginate cultures [72]. In addition, Khaleel and coworkers [73] reported the capability of resveratrol to sensitize CRC cells to DOX via facilitating apoptosis and enhancing the intracellular entrapment of DOX by blocking the activity of the P-gp pump. Interestingly, the same ability was also demonstrated by Didox, a synthetic polyphenolic compound that shares important biochemical targets with resveratrol [97]. Very recently, a novel strategy has been described in which nanoparticles filled with resveratrol or OXA were applied on in vitro systems. The combination of OXA and resveratrol nanoparticles exerted a synergistic effect, with a higher cytotoxicity than the nanoparticle alone or the free drugs, indicating this approach as a promising strategy for CRC therapy. In mice implanted with CT26 cells, the combination of OXA (8 mg/kg, intravenous (i.v.)) and resveratrol (30 mg/kg i.v.) every 2 days for 20 days reduced tumor size and the proportion of tumor-infiltrating myeloid-derived suppressor cells (MDSCs), thus impairing the immune escape ability of the tumor [74].

#### 4.1.3. Other Dietary Polyphenols

Quercetin is a major constituent of various dietary products whose cancer prevention and treatment potentials have been extensively explored [98,99]. A combination of quercetin and IR exhibited dramatic anticancer effects by targeting colon CSC and inhibiting Notch-1 signaling in vitro [75]. These effects were also confirmed in vivo in HT-29 xenografts receiving quercetin (10 mg/kg/day s.c.) and/or radiation (5 Gy/week) treatment for four weeks. The combined treatment significantly reduced tumor volume compared to controls and induced a remarkable decrease in CSC markers and Notch-1 signaling protein expression [75]. Although quercetin possesses great medicinal value, its use as a therapeutic agent is hampered by its poor oral bioavailability. The loading of quercetin onto self-assembling lecithin-stabilized polymeric micelles (LsbPMs) provided a greater stability of hydrophobic chemicals and an enhanced bioavailability as compared to free quercetin [76]. A combination of DOX (4 and 2 mg/kg i.v.) with this quercetin formulation (50 mg/kg i.v.) resulted in an efficient growth inhibition of CT26 colon cancer cells and reduced cardiotoxicity in vivo [76].

Bound polyphenol of inner shell (BPIS) from foxtail millet bran displayed effective antitumor activities in vitro and in vivo. BPIS significantly increased the sensitivity of human drug-resistant CRC cell lines to OXA. Of particular relevance is the ability to reverse the multidrug resistance of CRC when cells exposed to 5-FU, OXA and vincristine (VCR) are pretreated with BPIS. The inhibition of cell proliferation, the promotion of cell apoptosis and the accumulation of rhodamine-123 (Rh-123) in HCT-8/Fu cells are among the mechanisms exploited by BPIS to synergize with chemotherapy [77]. More recently, remodeling of ganglioside GM3 catabolism [78] and upregulation of the miR-149 expression [79] have been proposed as mechanisms underlying the reversal effect of polyphenols on cancer drug resistance.

In an attempt to find molecules to prevent the emergence of drug resistance, Riahi-Chebbi and coworkers [80] recently investigated the antitumoral effect of thirteen phenolic compounds, from the Tunisian quince (*Cydonia oblonga* Miller), on the human 5-FU-resistant LS174-R CRC cells either as monotherapy or in combination with 5-FU. Only kaempferol was able to chemosensitize 5-FU-resistant LS174-R cells, exerting a synergistic inhibitory effect on cell viability. Kaempferol has already shown anticancer effects in several cancer cell lines, while exhibiting almost no or minor toxicity against normal epithelial, peripheral blood and myeloid cells [100,101]. A combination of kaempferol with 5-FU impacted on different cellular effectors, finally enhancing apoptosis and cell cycle arrest [80]. In particular, this combination blocked the production of ROS; modulated the expression of JAK/STAT3, MAPK, PI3K/AKT and NF-κB [80]; and increased levels of the pro-apoptotic protein Bax while downregulating the anti-apoptotic protein Bcl-2 and thymidylate synthase [81], an enzyme involved in DNA replication.

Silybin, a flavonoid extracted from the milk thistle seeds of *Silybum marianum*, has been used in an attempt to limit regorafenib’s side effects, thus improving its tolerability and enhancing its clinical activity. Regorafenib is a very promising drug for chemorefractory mCRC patients, but its toxicity profile has limited its use in clinical practice. A combined treatment of silybin and regorafenib induced, in a panel of human colon cancer cell lines with distinct mutations in the KRAS, NRAS, BRAF, and PIK3CA genes, synergistic anti-proliferative and apoptotic effects via the inhibition of the PI3K/AKT/mTOR pathway and the production of ROS within cells [82].

In recent years, propolis has raised great interest due to a variety of pharmacological effects, including anticancer properties [102]. Propolis consists of a mixture of different compounds, including polyphenols, whose composition depends on the geographical site of production [102,103]. Administering an alcoholic extract of propolis (10, 30 or 90 mg/kg i.p., 5 times a week) rich in chrysin, galangin and pinocembrin in combination with 5-FU (50 mg/kg, once a week) for 8 weeks in AOM/DSS-induced CRC reduced the number of pathological lesions in comparison with 5-FU-based monotherapy through decreasing the expression of β-catenin, iNOS and COX-2 proteins in the intestinal tissue [83].

Several other polyphenolic compounds have been used in combination with 5-FU or OXA in in vitro assays, to potentiate its activity and to reduce the side effects, in an attempt to improve the therapeutic potential of the treatment. In this context, studies have been reported on tangeretin [84], schizandrin [85], luteolin [86], epigallocatechin gallate (EGCG) [87], the ohmic extract of vine pruning residue (VPE) [88] and even on dual drug loaded liposomes bearing apigenin and 5-FU [89]. These investigations reported an increase in the cytotoxic activity of 5-FU or OXA in combined exposure by stimulating different mechanisms influencing the life and death of the cells. Polydatin, a glycoside of resveratrol, decreased the radioresistance of colorectal CSC via the BMP signaling pathway, thereby sensitizing tumor cells to radiotherapy [90].

To summarize, the studies herein described provide evidence that combining treatments of natural polyphenols with anticancer drugs is a promising strategy able to improve the effectiveness of treatment and to overcome drug resistance.

### 4.2. Data from Clinical Trials

In light of the evidence obtained in preclinical models, a clinical trial evaluation is mandatory for translation into humans and to further validate the feasibility and efficacy of such natural product formulations for their use in a clinical setting. Of note, a de-creased total antioxidant capacity associated with a progressive reduction in polyphenol plasma levels has been described in stage II–IV CRC patients, with significantly lower values in subjects undergoing therapy with cytostatic drugs, alone or in combination with monoclonal antibodies [104].

As stated above, the efficacy of 5-FU-based chemotherapy in advanced, inoperable CRC patients is limited due to its intolerable adverse effects. A number of human studies have been registered with the aim of evaluating the adjuvant potential of polyphenols (mainly phenolic acids and flavonoids) to standard treatments for advanced/mCRC. However, to date, only a few of them have reported the outcome (Table 2), possibly due to still on-going investigations or discontinuation of the studies.

The CUFOX trial was the first study that combined daily oral curcumin with FOLFOX-based chemotherapy in subjects with a histological diagnosis of mCRC [105]. A phase I dose-escalation study was designed to determine the acceptable target dose of curcumin in order to safely proceed to a phase IIa open-labeled randomized controlled trial. The addition of daily oral curcumin to standard chemotherapy turned out to be safe and tolerable. No significant difference between FOLFOX and CUFOX (FOLFOX plus curcumin) arms was reported in terms of quality of life or neurotoxicity [106]. However, an increase in the median progression-free survival (PFS) and overall survival (OS) was achieved in CUFOX-treated patients with respect to the control group [106].

The clinical application of curcumin is greatly restricted due to its low water solubil-ity, poor oral absorption and rapid metabolism, and a number of curcumin nanoformula-tions have been developed to overcome these limitations. To date, three clinical trials on the use of curcumin nanoformulations in CRC and colorectal adenomatous polyposis have been registered in the ClinicalTrials.gov (last accessed on July 2021) database [54]. Among them, a phase II study was designed to investigate nanostructured lipid curcumin particles as a dietary supplement in combination with FOLFIRI/bevacizumab-based chemotherapy in patients with unresectable CRC liver metastases. The study included a two-year follow-up after the intervention for PFS and OS, with safety, quality of life and fatigue as secondary outcomes. However, no information on the outcome has been reported yet. More generally, although promising results have been generated from preclinical studies, only poor evidence has been provided that combining curcumin with chemotherapeutic agents is effective for treating cancer in humans [107].

Epidemiological and preclinical studies have demonstrated a role for genistein, a soy-derived isoflavon whose safety in humans has been well established, in colorectal malignancy prevention and treatment [56]. Recently, the first-in-human study of genistein in combination with FOLFOX or FOLFOX/bevacizumab therapy was approved for the upfront treatment of mCRC [108]. Subjects received up to six cycles of therapy every 2 weeks in combination with oral genistein. Therapy-responsive patients went on to complete six additional cycles or underwent surgical resection when feasible. The response rate and PFS obtained in this pilot study were substantially better than those previously reported for chemotherapy alone [109], suggesting that the combination treatment can improve efficacy.

A different flavonoid, fisetin, was studied for its potential adjuvant effect on CRC therapy, in virtue of its strong anti-inflammatory acitvity. Specifically, a clinical study was designed with the aim of assessing the efficacy of fisetin supplementation on the inflammatory status and matrix metalloproteinase (MMP) levels in CRC patients who had undergone primary tumor resection two weeks prior to the intervention [110]. In this double-blinded, randomized placebo-controlled clinical trial, stage II and III CRC patients undergoing chemotherapy (OXA infusion plus oral capecitabine) were assigned to receive either fisetin or placebo for seven consecutive weeks. Despite the short supplementation period, this study highlighted a beneficial effect of fisetin on patient inflammatory status, with a significant decrease in serum levels of C-reactive protein, IL-8 and MMP-7. This study also paved the way toward future clinical investigations aimed at determining the more specific effects of this flavonoid compound on disease outcome.

Polyphenols have also been explored for their possible use in CRC patients with progressive disease due to unresponsiveness to standard chemotherapies. In a prospective proof of concept study, mCRC patients were treated with regorafenib in combination with a complex of silybin, vitamin E and phospholipids, after failure of all available therapies [82]. In this formulation, silybin was found to increase the clinical efficacy and tolerability of regorafenib, leading to increased PFS and OS [82]. Silybin combined with regorafenib was suggested as a promising strategy to improve the efficacy of this recently approved drug, whose toxicity has limited its use in clinical practice.

The idea that polyphenols can act as adjuvants to CRC therapy is strongly supported by the evidence found in human studies. However, future studies involving a larger number of subjects and including control groups are recommended to move toward clinical application.

## 5. Conclusions

CRC is one of the deadliest types of cancer worldwide. By 2030, the mortality rate of CRC is expected to increase by 60%. Therapy failure, due to low efficacy, heavy adverse effects and drug resistance, is a major concern. Although dietary polyphenols have been extensively investigated for their chemopreventive and direct anticancer effects on CRC, the idea of using polyphenols in combination with standard treatments to increase efficacy and cope with therapy toxicity is extremely intriguing. Chemo-/radio-sensitization, autophagy inhibition, the harnessing of migration and immunomodulation are the key mechanisms ascribed to dietary polyphenols in combination with current CRC treatments to date. Nevertheless, the multiplicity of cell targets and molecular pathways activated by dietary polyphenols will require a careful preclinical evaluation of the host health status, disease stage and of the companion drugs before clinical translation.

The bioavailability and bioactivity of polyphenols are also critical issues, which largely rely on their formulation and on inter-individual variability. Differences in absorption, distribution and metabolism, as well as age, sex, genetics and epigenetic factors, all contribute to the inter-individual variability of response to polyphenols. It is worth noting that dietary polyphenols and/or their metabolites are present at low concentrations in the cells. If administered at very high concentrations, polyphenols may cause toxic effects. Indeed, adverse effects of polyphenols have been reported in some experimental studies [111,112], although it has not always been proven that these effects also occur in humans. In addition, the co-administration of natural products along with conventional medicines can modify their bioavailability and toxicity profile. Therefore, the vigilance for their potential dose-related toxicity should remain high. In addition, in virtue of their antioxidant properties, polyphenols may, on one hand, alleviate the adverse effects of chemotherapy and/or radiotherapy and, on the other hand, antagonize oxidative damage induced by these treatments. Studies on animal models have started to address these issues by exploring the best dosage, route of administration and formulation to optimize anticancer effects and minimize the detrimental effects on companion drugs. However, it is necessary to take into account that a beneficial dose in an animal model can be harmful when applied in a human setting. Alternatively, a dose used in an experimental study may never occur in humans because the bioavailability is very low or because the appropriate dose never reaches the target site.

While surgery and chemo- and radio-therapy interventions continue to represent essential treatments of CRC depending on the tumor stage, the experimental evidence reported herein highlights the enormous potential of dietary polyphenols as adjuvants of current anticancer strategies and paves the way for the further development of these compounds in the optimal therapeutic management of CRC patients.

## Figures and Tables

**Table 1:** Preclinical studies on the effects of dietary polyphenols in combination with standard or experimental CRC treatments.

Polyphenol	Combination with	In Vitro Model	In Vivo Model	Main Effects of the Combination	Ref
**Curcumin**	5-FU	SW620	-	↓ Proliferation and migration, ↓ pERK signaling, ↓ L1 expression *	[59]
	5-FU	HCT116, HT-29	HCT116 xenograft	↓ Tumor growth, ↓ autophagy	[60]
	IRI	LoVo, HT-29	-	ROS generation, ER stress, apoptosis, cell cycle arrest	[61]
	IRI	resistant LoVo	-	CSC apoptosis, ↓ CSC markers, ↓ chemoresistance	[62]
	OXA	resistant HCT116	-	↓ Drug resistance, ↓ p-p65 and Bcl-2, ↓ migration, ↑ caspase-3	[63]
	OXA	resistant HCT116	-	↓ Drug resistance through effects on miR-409-3p	[64]
	Regorafenib	HCT116	-	↓ Tumor growth, ↓ autophagy, apoptosis	[65]
	Sildenafil + 5-FU	-	CT26-implanted mice	↓ Tumor growth, ↑ DNA damage signaling, metabolic and endoplasmic reticulum stress signaling	[66]
	Sildenafil + anti-PD1	-	CT26-implanted mice	↓ Tumor growth	[66]
	GEM	Patient-derived HROC	-	Apoptosis, ↑ immune responses	[67]
	IR	HT-29	HT-29 xenografts	Apoptosis, altered expression of DNA repair-related genes, ↓ Tumor growth	[68]
**Curcumin analogs**	5-FU	SW480, SW620	-	Apoptosis, ↓ cell cycle, ROS production, ↓ MMP, ER expansion	[69]
	FOLFOX	HCT116	-	↓ Tumor growth, ↓ colonospheres formation	[70]
**Curcumin+Resveratrol Nanoformulation**	mEHT	CT26	CT26-implanted mice	↑ Immunogenic apoptosis, ↓ tumour growth, recruitment of T cells and F4/80 + macrophages	[71]
**Resveratrol**	5-FU	HCT116, resistant HCT116	-	↓ TNF-β-induced tumor metastasis, chemosensitization of CRC cells	[72]
	DOX	HCT 116, HT-29	-	Modulation of apoptotic proteins, cell cycle arrest, blocking of the efflux activity of p-gp	[73]
**Resveratrol nanoformulation**	OXA	SW480	CT26-implanted mice	↓ Tumor growth, ↓ immunosuppression	[74]
**Quercetin**	IR	HT-29, DLD-1	-	↓ Cell proliferation, apoptosis, ↓ Notch-1 signaling, ↓ CSC markers, ↓ toxicity toward normal cells	[75]
**Quercetin-loaded micelles**	DOX	-	CT26-implanted mice	↓ Tumor growth, ↓ cardiotoxicity	[76]
**BPIS**	5-FU, OXA, VCR	HCT-8/Fu	-	Multi-drug resistance reversal, ↓ cell proliferation, apoptosis, accumulation of rhodamine-123	[77]
	OXA	resistant HCT116,	-	↓ Cell proliferation, modeling ganglioside GM3 catabolism	[78]
	OXA	HCT-8/Fu		↓ Drug resistance, ↓ miR-149 methylation	[79]
**Kaempferol**	5-FU	resistant LS174-R	-	Apoptosis, cell cycle arrest	[80]
	5-FU	HCT-8, HCT116	-	↓ Cell proliferation and viability, apoptosis	[81]
**Sylibin**	Regorafenib	LoVo, HCT15, SW48, SW48-CR, GEO, GEO-CR, SW620, SW480, HCT116 LIM1215		↓ Cell proliferation, apoptosis, ROS generation	[82]
**Chrysin, Galangin and Pinocembrin from Propolis**	5-FU	-	AOM/DSS-treated mice	↓ Number of tumor lesions, ↓ β-catenin, iNOS, and Cox-2 protein expression	[83]
**Tangeretin**	5-FU	HCT116, HCT-15	-	Apoptosis, ROS generation, ↓ ATP, ↑ DNA damage	[84]
**Schizandrin**	5-FU	HCT116, SW480	-	Apoptosis, ↓ PI3K/AKT and NF-κB pathways, ↑ miR-195	[85]
**Luteolin**	OXA	HCT116	-	Apoptosis, ↑ Nrf2 activity	[86]
**EGCG**	5-FU	HCT116, DLD-1	DLD-1 xenograft	↓ Cell viability and colony formation, apoptosis, ↓ tumor growth	[87]
**Vine pruning residue**	5-FU	HCT116, RKO	-	↓ Cell proliferation, DNA effects and cell cycle modulation	[88]
**Apigenin**	5-FU	HCT15	-	Apoptosis	[89]
**Polydatin**	IR	CT26, Lgr5+ CSC, HCT116	AOM/DSS-treated mice	↓ Cell proliferation apoptosis, ↓ radioresistance via the BMP signaling pathway, ↓ CRC proliferation, ↓ tumor number and volume, ↑ survival	[90]

Abbreviations: 5-FU, 5-fluorouracil; IRI, irinotecan; DOX, doxorubicin; OXA, oxaliplatin; VCR, vincristine; mEHT, modulated electro-hyperthermia; IR, ionizing irradiation; EGCG, epigallocatechin gallate; BPIS, bound polyphenol of inner shell; GEM, gemcitabine. * It is mentioned in the following text.

**Table 2:** Clinical studies evaluating the effects of polyphenols and standard therapy combination in CRC patients.

Polyphenol (Dose)	Subjects/Study Type	Experimental Group	Control Group	Main Outcome	Refs/Trial
Curcumin (C3 complexed, 2 g/day)	Stage IV mCRC pts/Phase IIa randomized controlled	FOLFOX + Curcumin (CUFOX, *n* = 18)	FOLFOX (*n* = 9)	↑ PFS ↑ OS = QOL ↓ neuropathy scores (trend)	Clinicaltrials.gov (last accessed in June 2021) NCT01490996 (completed) [83,84]
Curcumin (nanostructured lipid particles, 100 mg twice/day)	Stage IV mCRC pts/Interventional Phase II	FOLFIRI/Bevacizumab + Curcumin (*n* = 50)	None	In progress	Clinicaltrials.gov (last accessed in June 2021) NCT02439385 (recruitment completed, 2020)
Genistein (60 mg/day)	Stage IV mCRC pts/Interventional Phase I/II Pilot	FOLFOX + Genistein (*n* = 10) FOLFOX/Bevacizumab + Genistein (*n* = 3)	None	↑ RR ↑ PFS	Clinicaltrials.gov (last accessed in June 2021) NCT01985763 (completed, 2018) [87]
Fisetin (100 mg/day)	Stage II/III CRC pts/Randomized placebo controlled	OXA infusion/oral CAPE + Fisetin (*n* = 18)	OXA infusion/oral CAPE + placebo (corn starch, *n* = 19)	↓ serum CRP, IL-8, MMP-7	www.irct.ir (last accessed in June 2021) IRCT2015110511288N9 [89]
Silybin (188 mg/day, complexed with PC and vitamin E)	Pre-treated advanced mCRC pts/Prospective Pilot	Regorafenib + Silybin (*n* = 22)	None	↑ PFS ↑ OS ↓ drug-induced liver damage	[75]

Abbreviations: pts, patients; FOLFOX, 5-fluorouracile, folinic acid and oxaliplatin; CUFOX, FOLFOX + curcumin; OXA, oxaliplatin; CAPE, capecitabine; PC, phosphatidylcholine; PFS, progression-free survival; OS, overall survival; RR, response rate; QOL, quality of life; CRP, C-reactive protein.
